# Next-Generation Sequencing in the Diagnostic Workup of Neonatal Dried Blood Spot Screening in Sweden 2015–2023

**DOI:** 10.3390/ijns11030073

**Published:** 2025-09-03

**Authors:** Lene Sörensen, Jorge Asin-Cayuela, Michela Barbaro, Helene Bruhn, Martin Engvall, Nicole Lesko, Karin Naess, Mikael Oscarson, Yan Shen, Malin Ueberschär, Anna Wredenberg, Fredrik H. Sterky, Anna Wedell, Rolf H. Zetterström

**Affiliations:** 1 Centre for Inherited Metabolic Diseases, Karolinska University Hospital Solna, 171 76 Stockholm, Sweden; 2 Department of Molecular Medicine and Surgery, Karolinska Institutet, 171 77 Stockholm, Sweden; 3 Department of Laboratory Medicine, Institute of Biomedicine, University of Gothenburg, 413 90 Gothenburg, Sweden; jorge.asin.cayela@vgregion.se (J.A.-C.);; 4 Department of Clinical Chemistry, Sahlgrenska University Hospital, 413 45 Gothenburg, Sweden; 5 Department of Medical Biochemistry and Biophysics, Karolinska Institutet, 171 77 Stockholm, Sweden

**Keywords:** newborn screening program, next generation sequencing, whole genome sequencing

## Abstract

Sweden has one neonatal screening laboratory and two centers conducting diagnostic workup for inborn errors of metabolism (IEM). Next-generation sequencing (NGS) has been gradually introduced as a confirmatory diagnostic test in the Swedish newborn screening program. Here, we describe the use of NGS in the diagnostic workup of IEM in screening-detected babies in Sweden between 2015 and 2023. During this period, 1,023,344 newborn children were screened, and 81 of 290 IEM cases were genetically confirmed using NGS. Planned improvements to the program are to perform genetic validation directly on the initial dried blood spot (DBS). As whole-genome sequencing (WGS) is superior in detecting causative genetic variants compared to Sanger sequencing, targeted NGS, and whole-exome sequencing (WES), it will likely become the method of choice more broadly in the future. A strong focus is to consolidate the nationally coordinated DBS newborn screening program, with all its individual components, including screening, targeted diagnostics, individualized treatment, and follow-up. This challenges the current regionalized organization of Swedish healthcare, which hinders close national collaboration between experts and sharing of data, as well as equal access to advanced treatments for identified patients, regardless of their place of birth.

## 1. Introduction

It has been more than 60 years since population-wide newborn screening began in 1963 in Oregon and Massachusetts, U.S.A., following Robert Guthrie’s pioneering work [1]. Sweden followed in 1965 [2] and currently screens for 26 different conditions, 22 of which are inborn errors of metabolism (IEM) (Table 1).

Sweden has one neonatal screening laboratory, the PKU laboratory at the Centre for Inherited Metabolic Diseases (CMMS), Karolinska University Hospital, Stockholm, receiving samples from around one hundred thousand newborns per year. CMMS is built as an integrated center, hosting the Swedish dried blood spot (DBS) newborn screening laboratory (the “PKU laboratory”), together with facilities and expertise for biochemical, genetic, and clinical investigations and long-term follow-up of IEM. Diagnostic workup of IEM, including follow-up of screening-positive cases, is carried out at two centers: CMMS in Stockholm and the Department of Clinical Chemistry at Sahlgrenska University Hospital in Gothenburg. National, long-term follow-up of IEM patients is performed at three specialized pediatric centers and supported through the national quality registry for inborn errors of metabolism (RMMS.se).

Since the start of PKU, newborn screening programs have expanded successively. For a recent and very comprehensive review, see the work by Therrell and coworkers [3]. Initially, new tests needed to be developed each time a new condition was added. In the 1990s, a major methodological advance was achieved with electrospray ionization linked to tandem mass spectrometry, first proposed by Millington and colleagues [4], which enabled the simultaneous detection of up to 60 disorders in a single test. Next-generation sequencing (NGS) is a game-changing technology that is revolutionizing the genetic diagnostics of rare diseases. Major efforts have been made in Sweden to implement genome sequencing for the diagnosis of rare diseases in healthcare—not primarily aiming for newborn screening, but with clear implications for the diagnostic workup of screening-detected babies. The Swedish clinical–academic landscape is, however, complex, making the clinical implementation of rare disease genomics complicated.

Swedish public healthcare is decentralized across 21 regions and financed primarily through taxes levied at the same level. Public funding for research and innovation, on the other hand, is a governmental responsibility. Genome sequencing requires infrastructure and expertise on a level beyond the scope of public healthcare funding and is thus critically dependent on academia. Science for Life Laboratory (SciLifeLab) is a national academic infrastructure funded by the Swedish government with the mission to provide high-throughput bioscience through technical platforms, including NGS.

To enable the integration of genomics into rare disease healthcare, the Genomic Medicine Center Karolinska–Rare Diseases (GMCK–RD) was established as an academic–clinical partnership between the SciLifeLab Clinical Genomics facility and public healthcare in the Stockholm region, using the diagnosis of patients with IEM as a pilot. Targeted whole-exome sequencing (WES) using in silico gene panels was initially used and replaced with whole-genome sequencing (WGS) using custom-designed in silico gene panels in 2015 [5,6]. For deep clinical integration, the multidisciplinary environment at CMMS has been essential, where experienced clinicians work side by side with experts in laboratory medicine, genomics, and bioinformatics. The concept has successively spread to additional disease groups through a close collaboration with the departments of Clinical genetics and Genomics, and Clinical Immunology, enabling targeted, customized analyses, as well as sharing and interpretation of genomic data across a broad range of rare disease groups. During the first five years of clinical genome sequencing, 3219 patients were investigated, and 1285 received specific diagnoses through this collaborative effort [6]. GMCK–RD is a regional node within the national Genomic Medicine Sweden initiative (GMS), which aims to spread genomic medicine nationally.

In Gothenburg, the Department of Clinical Chemistry at Sahlgrenska University Hospital has long been committed to the diagnosis and management of IEMs, integrating biochemical and genetic investigations in close collaboration with specialized clinicians. To keep pace with advances in genetic diagnostics, the Center for Medical Genomics (CMG) was established to consolidate analytical platforms for both clinical genetic diagnostics and research. Since 2019, CMG has offered whole-genome sequencing (WGS) as the standard platform for genetic diagnostics of IEM.

Here, we describe the results of using NGS in the diagnostic workup of IEM in screening-detected babies in Sweden between 2015 and 2023, at CMMS, Stockholm, as a part of GMCK-RD, and in Gothenburg, respectively.

## 2. Materials and Methods

### 2.1. Study Subjects

Children who had abnormal screening results for any of the 22 IEMs and who were referred for diagnostic workup, between 1 January 2015 and 31 December 2023, were included in this study.

### 2.2. Newborn Screening

For screening laboratory workflow, screening methodology, and data collection in the screening laboratory, see [7].

### 2.3. Biochemical and Genetic Confirmation

Diagnostic samples and a second DBS sample were collected, and clinical evaluation of the infant was performed at one of the metabolic treatment centers (five at the start of this study, now three) or at the home hospital, in close collaboration with a pediatric metabolic specialist, depending on the diagnosis and the clinical situation. The second DBS is used to confirm the identity of the child, provide feedback to the screening laboratory that the child has been taken care of and action has been taken, and provide guidance in the diagnostic workup. For an overview of the Swedish IEM DBS NBS system, see [7].

Positive screening cases were genetically confirmed unless biochemical diagnostic workup tests were conclusively negative, in the case of siblings with obvious biochemical and/or clinical phenotypes, or if prenatal testing had already been performed. Genetic investigations using DNA prepared from EDTA blood were performed with Sanger sequencing, WES, or WGS. Significance of variants was interpreted using ACMG criteria in combination with supporting clinical and biochemical data. At Karolinska, WGS has been used since 2015 for disorders where at least two candidate genes (Table 2), or for single-gene diseases such as CIT1 and ASL, for which Sanger sequencing had not been established, using methodology previously described in [6]. WGS is also used for cases in which Sanger sequencing was unable to identify two pathogenic variants in PAH if a child is referred for PKU. Briefly, WGS data were processed using the Mutation Identification Pipeline framework (MIP). The current version, MIP 8.2 (https://github.com/Clinical-Genomics/MIP, accessed on 21 July, 2025), detects single-nucleotide variants (SNVs), insertions and deletions (INDELs), structural variants, uniparental disomy (UPD), and repeat expansions. Variant interpretation was performed using our custom-developed decision support and data-sharing system, Scout. An in silico gene panel, WGS panel NBS IEM, was established, covering all genes known to cause any of the IEM included in the newborn screening program.

For patients investigated in Gothenburg, two different NGS strategies have been used. Between July 2017 and July 2019, genetic investigations were carried out with a custom-made gene panel (SureSelect^QXT^, Agilent Technologies, Santa Clara, CA, USA), essentially as described [8]. The panel targeted coding exons ±25 flanking bases of 37 genes. In one inconclusive case, a second sample was sent for WGS. Since July 2019, WGS followed by in silico filtering of gene(s) of interest has been used instead, irrespective of the number of candidate genes. Library preparation (TrueSeq DNA PCR-free, Illumina, San Diego, CA, USA) and sequencing (NovaSeq 6000, Illumina) were carried out at CMG, and bioinformatic analyses were conducted at the SciLifeLab Clinical Genomics facility in Gothenburg. DNA-scope (Sentieon, San José, CA, USA) was used for mapping and detection of single-nucleotide variants and indels, while CANVAS (Illumina) and Integrative Genomics Viewer were used for copy number variation analysis. Alissa Interpret software (Agilent Technologies) was used for variant filtering and interpretation. Sanger sequencing was used to verify some WGS findings, but primarily to genotype parents in anticipation of genetic counselling and predictive testing.

### 2.4. Compliance with Ethical Standards

The publication of this study was approved by the Swedish Ethical Review Authority; Approval Number 2019-05816; approval date, 18 December 2019.

## 3. Results

From 1 January 2015 to 31 December 2023, 1,023,344 newborn children in Sweden were screened. Screening outcomes, including positive predictive values (PPVs), for the 21 IEMs where NGS was used to resolve cases in Sweden during the study period, are presented in Table 3.

Out of 296 IEM cases, 82 were genetically confirmed using NGS, as described in Methods. At Karolinska, one case was resolved using WES, and 39 cases were resolved using WGS. At Sahlgrenska, 14 cases were resolved using the Agilent panel, and 29 cases were resolved using WGS. One case was resolved using NGS at Skåne University Hospital in Lund, making a total of 83 cases resolved using NGS in Sweden during this period. The distribution of genetically confirmed cases using NGS in Stockholm or Gothenburg is presented in Table 4.

## 4. Discussion

NGS has been gradually introduced as a confirmatory diagnostic test in the Swedish newborn screening program. In Stockholm, targeted IEM diagnostics have been intimately linked to the initial screening test for many years, using Sanger sequencing for validation. Sanger is therefore still the method of choice for most NBS diseases in uncomplicated cases where only one gene is suspected, whereas WGS has been used since 2015, when two or more genes can be causative. In Gothenburg, all NBS cases have been resolved using WGS since 2019. As WGS detects more genetic variants, this technique is also used to resolve cases in which Sanger sequencing is unable to identify two pathogenic variants. It is, however, clear from our experience as well as from other studies that WGS is superior in detecting causative genetic variants compared to both Sanger sequencing and WES, and it will likely become the method of choice more broadly in the future. However, for certain disorders, Sanger sequencing can still be the first choice and a good complement to other NGS methods, as already demonstrated in screening algorithms for single-gene diseases [9]. Examples resolved by WGS from the current study include deep intronic variants in *ASS1* causing citrullinemia, deletion of a complete exon in *ARG1* causing arginase deficiency, and uniparental disomy with homozygous pathogenic variants in *PCCA* and *HADHA*, causing propionic aciduria and LCHAD deficiency, respectively. One child affected with both CPT2 and MAD deficiencies was also identified, caused by bi-allelic variants in both *CPT2* and *FLAD1*. Analysis of one patient with tyrosinemia type I found only a heterozygous SNV using the Agilent exon-based panel, whereas subsequent analysis using WGS revealed the presence of a 50.7 Mb inversion with a breakpoint in intron 11 of *FAH*.

Swedish healthcare is currently going through structural changes, supporting the concept of multidisciplinary, integrated diagnostics and facilitating national coordination. The Swedish National Board of Health and Welfare coordinates a process in which specific areas are concentrated into a few units with national responsibility (National specialized medical care units). From July 2024, the PKU laboratory at CMMS in Stockholm will be the only unit authorized to perform newborn screening on DBS. Two units, in Stockholm and Gothenburg, are authorized to perform targeted diagnostics for IEM, based on their combined genetic/genomic and biochemical expertise, as well as long-standing clinical competence and experience of IEM. Three units, Stockholm, Gothenburg, and Lund, will be responsible for advanced follow-up and treatment of IEM. All units use the national quality registry RMMS for long-term follow-up, providing feedback to the PKU laboratory and enabling continuous monitoring of quality parameters. A corresponding national centralization has been implemented for neuromuscular diseases, for which two sites are now primary referral centers for spinal muscular atrophy (SMA) DBS NBS. The area of primary immunodeficiency (PID), including severe combined immunodeficiency (SCID), which is also included in the NBS program, is currently going through the same process. It has recently been decided that treatment and follow-up of PID disorders will be centralized to three treatment centers (the same sites as for IEMs). The Swedish DBS NBS program, comprising screening, targeted diagnostics, treatment, and long-term follow-up, will thus be consolidated and held together at the national level. This will provide a solid basis for maintaining precision and quality, which is important for future expansion.

A planned improvement of the program is to perform genetic validation directly on the initial DBS. This is expected to reduce false positives, ensure uniform and rapid genetic validation, and shorten the time required for diagnostic workup [10,11,12,13,14]. A second DBS will still be used, as described in the Section 2.

NGS has the potential to transform clinical medicine in many ways, and efforts to use genomic testing as a first-tier test in newborn screening are planned or underway in several centers worldwide [15] (https://www.iconseq.org/global-projects, accessed on 21 July 2025). The Genomics England research project aims to screen 100,000 newborns for treatable monogenic disorders using WGS as an adjunct to the current screening program, starting in 2023 and aiming for more than 200 genetic disorders (https://www.genomicsengland.co.uk/initiatives/newborns/ethics, accessed on 21 July 2025). Screen4Care is a 5-year-long European Project, launched in 2021 and funded under the Innovative Medicine Initiative. In a pilot study, 20,000 newborns will be screened using either one or two panels: one with around 80 conditions defined as “treatable” and the other with around 200 conditions, including 120 conditions for which there is no known treatment or clinical interventions, but which are considered “actionable” (www.screen4care.eu, accessed on 21 July 2025).

Newborn screening using genomics as a first-tier test differs drastically from classical newborn screening programs, which rely on functional first-tier tests combined with targeted diagnostic workup, including genomics. Most affected babies detected through DBS screening programs worldwide have congenital hypothyroidism, which is not suitable for genetic screening, as most of them are not monogenic. A large study comparing WES with traditional biochemical testing also demonstrated the limitations of using genetics as a first-tier test in NBS for IEMs [16]. Interpreting genetic variants without a functional readout can be very difficult, and only a proportion of clearly harmful variants can safely be considered as disease-causing. If used as a first-line test in newborn screening, sensitivity would thus have to be low to avoid false positives and uncertain results. Returning uncertain findings could cause overdiagnosis and anxiety in families as well as unnecessary burdens on the healthcare system. Broadening the set of diseases tested, particularly those with less clear benefits, may lead more parents to opt out of testing altogether. This could risk current screening programs by resulting in missed cases of, e.g., congenital hypothyroidism, the single most common condition included.

New treatments, however, offer the potential for healthier lives for children affected by previously untreatable diseases, and for some diseases, first-tier genetic screening may be motivated, provided that appropriate measures are taken. It will be essential to follow developments in this rapidly developing area, learn from ongoing activities, and carefully explore their harms and benefits.

As described, we are focusing on promoting a nationally coordinated newborn screening program. Significant advances have been made, such as the formation of national care units (NHVs), which are partly compatible with European reference networks, such as MetabERN. However, several issues remain to be solved to consolidate this successful concept on the national level. These include guaranteeing a sustainable infrastructure for the current system with all its individual components, including screening, targeted diagnostics, individualized treatment, and follow-up. The current regionalized organization of Swedish healthcare thus needs to be challenged to enable close collaboration between critical expertise and sharing of data on a national level, as well as provide equal access to advanced treatments for identified patients, regardless of their place of birth.

## Figures and Tables

**Table 1:** The 26 screening diseases currently included and evaluated by the National Board of Health and Welfare using dried blood spots (DBSs).

Disease Group	Disease	Year Added
Endocrine disorders	CAH	1986
	CH	1980
Aminoacidopathies	HCY	2010
	MSUD	2010
	PKU	1965
	TYR1	2010
Carnitine disorders	CACT	2010
	CPT1	2010
	CPT2	2010
	CUD	2010
Fatty acid oxidation defects	LCHADD	2010
	MADD	2010
	MCADD	2010
	VLCADD	2010
Organic acidurias	BKT2	2010
	GA1	2010
	IVA	2010
	MMA	2010
	PA	2010
Urea cycle defects	ARG	2010
	ASL	2010
	CIT1	2010
Others	BIOT	2002
	GALT	1967
	SCID	2019
	SMA	2023

**Table 2:** List of IEMs with corresponding candidate genes for which WGS is used to identify pathogenic variants in Stockholm. The Gothenburg site currently resolves all IEMs using WGS, but in Stockholm, Sanger sequencing is used primarily to resolve PKU, IVA, GA1, Tyr1, BIOT, GALT, HCY, MCADD, VLCADD, and CUD. BKT2 has not been resolved using NGS during the study period.

Disease Group	Disease(s) ^1^	Gene(s)
Aminoacidopathies	MSUD	*BCKDHA* *BCKDHB* *DBT* *DLD* *PPM1K*
	PKU/BH4-deficiency	*DNAJC12* *GCH1* *PAH* ^2^ *PCBD1* *PTS* *QDPR*
Carnitine disorders	CACT/CPT2	*CPT2* *SLC25A20*
	CPT1	*CPT1A*
Fatty acid oxidation defects	LCHADD/TFP	*HADHA* *HADHB*
	MADD	*ETFA* *ETFB* *ETFBH* *FLAD1* *SLC52A1* *SLC52A2* *SLC52A3*
Organic acidurias	MMA/CBLA/CBLB/CBLC/ CBLD/CBLF/CBLJ	*ABCD4* *LMBRD1* *MCEE* *MMAA* *MMAB* *MMACHC* *MMUT*
	PA	*PCCA* *PCCB*
Urea cycle defects	ARG	*ARG1*
	ASL	*ASL*
	CIT1	*ASS1*

^1^ Includes other diseases identified through the present screening algorithm (secondary conditions). ^2^ WGS is used in the case of PAH if Sanger sequencing fails to resolve the pathogenic genetic variant(s).

**Table 3:** All referrals due to abnormal screening tests, true positive cases, false positive cases, known missed cases, incidences, and PPVs for the 21 IEMs where NGS was used to resolve cases in Sweden during the 2015–2023 study period.

Disease Group	Disease(s) ^1^	All Referrals	True Positive Cases	False Positive Cases	Known Missed Cases	Incidence	PPV
Aminoacidopathies	HCY	9	3	6	0	1:340,000	33%
	MSUD	13	8	5	0	1:130,000	62%
	PKU/BH4-deficiency	72	71	1	0	1:14,000	99%
	TYR1	14	14	0	0	1:73,000	100%
Carnitine disorders	CACT/CPT2	18	5 ^2^	13	0	1:200,000	27%
	CPT1	3	2	1	0	1:510,000	67%
	CUD	116	13	103 ^3^	0	1:79,000	11%
Fatty acid oxidation defects	LCHADD/TFP	10	9	1	0	1:100,000	90%
	MADD	52	9^2^	43	0	1:110,000	17%
	MCADD	58	53	5	0	1:19,000	91%
	VLCADD	45	20	25	1	1:50,000	44%
Organic acidurias	GA1	22	8	14	0	1:130,000	36%
	IVA	10	9	1	0	1:100,000	90%
	MMA/PA/CBLA/CBLC/CBLJ	110	13	97 ^4^	0	1:79,000	12%
Urea cycle defects	ARG	3	2	1	0	1:510,000	67%
	ASL	7	7	0	1	1:150,000	100%
	CIT1	28	11	17	0	1:93,000	39%
Others	BIOT	33	27	6	0	1:38,000	82%
	GALT	21	12	9	0	1:85,000	57%

^1^ Includes other diseases identified through the present screening algorithm (secondary conditions). ^2^ One child was a true positive for both CPT2 and MADD. ^3^ Includes maternal CUD. ^4^ Includes dietary B12 deficiency.

**Table 4:** All IEM cases detected and genetically verified at the two sites in Sweden during the 2015–2023 study period.

Disease Group	Disease(s) ^1^	Gene(s)	Stockholm		Gothenburg	
			WES	WGS	Agilent	WGS
Aminoacidopathies	HCY	*CBS*				1
	MSUD	*BCKDHA*		2		
		*BCKDHB*		3		
		*DBT*		2		
	PKU/BH4-def	*PAH*			3	5
		*PTS*		2		1
	TYR	*FAH*			3	6
Carnitine disorders	CPT1	*CPT1A*		1		
	CPT2	*CPT2*		2		
		*CPT2 + FLAD1*		1^2^		
	CUD	*SLC22A5*				3
Fatty acid oxidation defects	LCHADD/TPF	*HADHA*		3	1	
		*HADHB*		2		
	MADD	*ETFA*		2		
		*ETFB*		1		
		*ETFDH*		3		
		*FLAD1 + CPT2*		1 ^2^		
	MCADD	*ACADM*			3	3
	VLCADD	*ACADVL*			1	
Organic acidurias	GA1	*GCHD*			1	1
	IVA	*IVD*				1
	MMA/CBLA/CBLB/CBLC/CBLJ	*ABCD4*				1
		*MMAA*		1		
		*MMAHC*		1		
		*MMUT*		4	1	1
	PA	*PCCA*		2		
		*PCCB*		1		
Urea cycle defects	ARG	*ARG1*		2		
	ASL	*ASL*		2		1
	CIT1	*ASS1*	1	1	1	1
Others	BIOT	*BTD*				3
	GAL	*GALT*				1
Total			1	39	14	29

^1^ Includes other diseases identified through the present screening algorithm (secondary conditions). ^2^ This patient had two confirmed homozygous variants, making it positive for both CPT2 and MAD.

## Data Availability

The original contributions presented in this study are included in the article. Further inquiries can be directed to the corresponding author.

## Abbreviations

The following abbreviations are used in this manuscript:

ARG	Arginase deficiency
ASL	Argininosuccinate lyase deficiency
BH4-deficiency	Tetrahydrobiopterin deficiency
BIOT	Biotinidase deficiency
BKT2	Betaketothiolase deficiency 2
CACT	Carnitine acylcarnitine translocase deficiency
CAH	Congenital adrenal hyperplasia
CBLA	Cobalamin A deficiency
CBLC	Cobalamin C deficiency
CBLD	Cobalamin D deficiency
CBLF	Cobalamin F deficiency
CBLJ	Cobalamin J deficiency
CH	Congenital hypothyroidism
CIT1	Citrullinemia type 1
CMG	Center for Medical Genomics
CMMS	Centre for Inherited Metabolic Diseases
CPT1	Carnitine palmitoyl transferase 1 deficiency
CPT2	Carnitine palmitoyl transferase 2 deficiency
CUD	Carnitine uptake deficiency
DBS	Dried blood spot
DNA	Deoxyribonucleic acid
EDTA	Ethylenediaminetetraacetic acid
GA1	Glutaric aciduria type 1
GALT	Galactosemia
GMCK–RD	Genomic Medicine Center Karolinska–Rare Diseases
GMS	Genomic Medicine Sweden
HCY	Homocystinuria
IEM	Inborn errors of metabolism
INDELs	Insertions and deletions
IVA	Isovaleric aciduria
LCHADD	Long-chain 3-hydroxyacyl-coenzyme A dehydrogenase deficiency
MADD	Multiple acyl-coenzyme A dehydrogenase deficiency
MCADD	Medium-chain acyl-coenzyme A dehydrogenase deficiency
MetabERN	European Reference Network for Metabolic Disorders
MIP	Mutation Identification Pipeline
MMA	Methylmalonic aciduria
MSUD	Maple syrup urine disease
NGS	Next generation sequencing
NHV	National care units (in Sweden)
PA	Propionic aciduria
PID	Primary immuno deficiency
PKU	Phenylketonuria
PPV	Positive predictive value
RMMS	Swedish national registry for inherited metabolic diseases
SCID	Severe combined immunodeficiency
SciLifeLab	Science for Life Laboratory
SMA	Spinal muscle atrophy
SNVs	Single nucleotide variants
TFP	Trifunctional protein deficiency
TYR1	Tyrosinemia type 1
UDP	Uniparental disomy
VLCADD	Very long-chain acyl-coenzyme A dehydrogenase deficiency
WES	Whole exome sequencing
WGS	Whole genome sequencing
